# The Prevalence of Atopic Dermatitis and Food Allergy in Children Living in an Urban Agglomeration—Is There a Current Relationship?

**DOI:** 10.3390/jcm12185982

**Published:** 2023-09-15

**Authors:** Marcel Mazur, Wojciech Dyga, Ewa Czarnobilska

**Affiliations:** Department of Clinical and Environmental Allergology, Jagiellonian University Medical College, Botaniczna St. 3, 31-501 Krakow, Poland

**Keywords:** epidemiology, atopic dermatitis, food allergy, air pollution

## Abstract

Atopic dermatitis (AD) prevalence in Poland is more frequent in individuals who live in a city. There are more studies demonstrating that long-term exposure to air pollutants is an independent risk factor for developing AD. The aim of the study was to assess the epidemiology of AD and food allergy (FA) in school children and adolescents living in Krakow, and to find a potential relationship between the incidence of atopic dermatitis with exposure to polluted air. In this paper, we presented the incidence of AD and FA between 2014 and 2018. We analyzed data collected from nearly 30,000 children aged 7–8 and adolescents aged 16–17 from the population of children and youth in Krakow. We correlated it with annual mean concentrations of PM10 and PM2.5, which indicated a gradual improvement in the air quality in Krakow. As our research results show that the prevalence of atopic dermatitis decreased with food allergy prevalence depending on the age group. We can suspect that this is the result of children growing out of a food allergy. It may be also influenced by more consequential eating habits in a group of adolescents and the elimination of allergenic foods from the diet. The decreasing incidence of atopic dermatitis appears to be also related to improvement in air quality.

## 1. Introduction

Allergy is a civilization disease. It was observed that city residents are particularly vulnerable. The prevalence of allergic diseases, especially among children, has become an increasing problem in the last few decades [1,2].

Atopic dermatitis is a chronic inflammatory disease that can manifest with different clinical phenotypes, causing diagnostic difficulties and requiring different therapeutic strategies. However, limited data exist regarding treatment in relation to individual clinical phenotypes [3].

AD prevalence in Poland is below the average for Europe (3.9% vs. 10%); however, the risk factors are similar to other countries. AD is more frequent in individuals with atopic parents and high socioeconomic status living in a city [3,4].

To date, little evidence is available to determine whether AD can be caused by exposure to air pollutants, including gases and particulate matter. However, there are more studies demonstrating that long-term exposure to air pollutants, including gases and particulate matter, is an independent risk factor for developing AD [5].

The aim of the study was to assess the epidemiology of AD and food allergy (FA) in school children and adolescents living in Krakow, the second largest urban agglomeration in Poland and to find a potential relationship between the incidence of AD with exposure to polluted air.

Clinical studies have documented the prevalence of FA in AD from 20% to 80% [6]. Many studies have indicated that FA plays an important role in exacerbating severe forms of AD and diet elimination will decrease the severity [7]. A high proportion of children with atopic dermatitis exhibit asymptomatic sensitization to foods [8]. Increasing evidence supports that AD predisposes patients to FA and not the opposite, while food allergens are presumed as one of the eliciting factors of AD exacerbations [9].

Therefore, we were interested in finding an answer to the question whether exposure to polluted air could explain the observed incidence of atopic dermatitis, and thus the risk of food allergy in the examined population.

## 2. Materials and Methods

The study participants lived and studied in Krakow during the study (2014–2018). All primary and secondary schools in Krakow were included in the study and all students in the studied age range were invited to participate in the study. The parents of students who consented to the participation of their children in the study and adolescent participants themselves were provided with questionnaires distributed to the students by school nurses and collected by them after filling.

The questionnaire was based on the International Study of Asthma and Allergy in Childhood (ISAAC) questionnaire translated from English to Polish by a professional translator, and included questions about episodes of eczema, an itchy rash located on the skin of the elbow and knee bends, ankles, buttocks, neck, ears or eyelids supplemented with questions about the development of an allergic reaction after eating certain foods [10]. The study, similarly to ISAAC, included children from certain age groups, including 7- and 8-year-olds and 16- and 17-year-olds.

The survey also included a request for parental (own and parental in the case of adolescents) consent for diagnostic tests, so that further on, students with a positive allergological history confirmed with completed questionnaires were referred to allergology units to conduct targeted allergological tests and consultations with an allergist. Based on the result of the questionnaire, supplemented with a medical history and physical examination, a preliminary initial diagnosis was established.

To estimate the prevalence of allergies to the most popular food allergens in the population of children and adolescents, the ImmunoCAP fx5 panel with food allergens including a mixture of egg white, milk, fish, wheat, peanut, and soybean allergens (Phadia AB, Uppsala, Sweden) was used.

After analyzing the results of the objective allergy tests, an ultimate diagnosis was made at the second consultation appointment.

FA and AD prevalence were matched with publicly available data from the Chief Inspectorate for Environmental Protection in Poland including annual mean concentrations of suspended particulate matter (PM), including coarse (PM10) and fine (PM2.5) particles, in Krakow obtained from three measurement points (µg/m3) in the years 2010–2018.

The study was conducted according to the guidelines of the Declaration of Helsinki, and approved by the Ethics Committee of Jagiellonian University (KBET/23/B/2014).

### Statistical Analyses

All the calculations were performed with Statistica 13 (TIBCO Software Inc., Palo Alto, CA, USA). The significant differences between the groups were determined using a chi-square test. Trend lines were fitted on the base of linear regression. Spearman’s test was used to measure the relationship between two variables. *p* values of <0.05 were considered statistically significant.

## 3. Results

The study covered 29,872 students in two age groups: 7–8 years (16,623 participants) and 16–17 years (13,249 participants). The study was based on questionnaires in which at least one positive answer concerning allergy symptoms was given (7732 children and 4583 adolescents); 2829 participants whose parents declared their will to continue participating qualified for the latter part of the study (fx5 panel with food allergens).

### 3.1. Results in the Group of Children 7–8 Years

**Figure 1:**
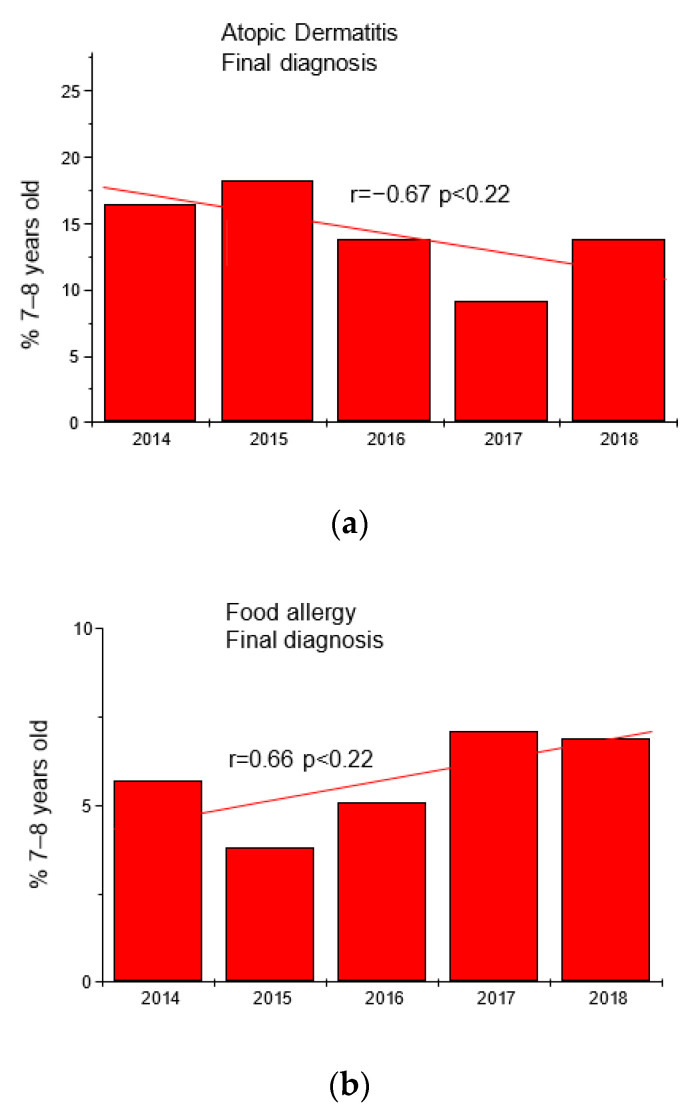
Trends in the frequency of diagnoses of atopic dermatitis (panel (**a**)) and food allergy (panel (**b**)) in the group of children 7–8 years in the studied years.

Based on the results of questionnaires and interviews, additional tests were performed in 2066 children (fx5 panel with food allergens). The results are shown in Table 1 and Figure 1.

### 3.2. Results in the Group of Adolescents 16–17 Years

**Figure 2:**
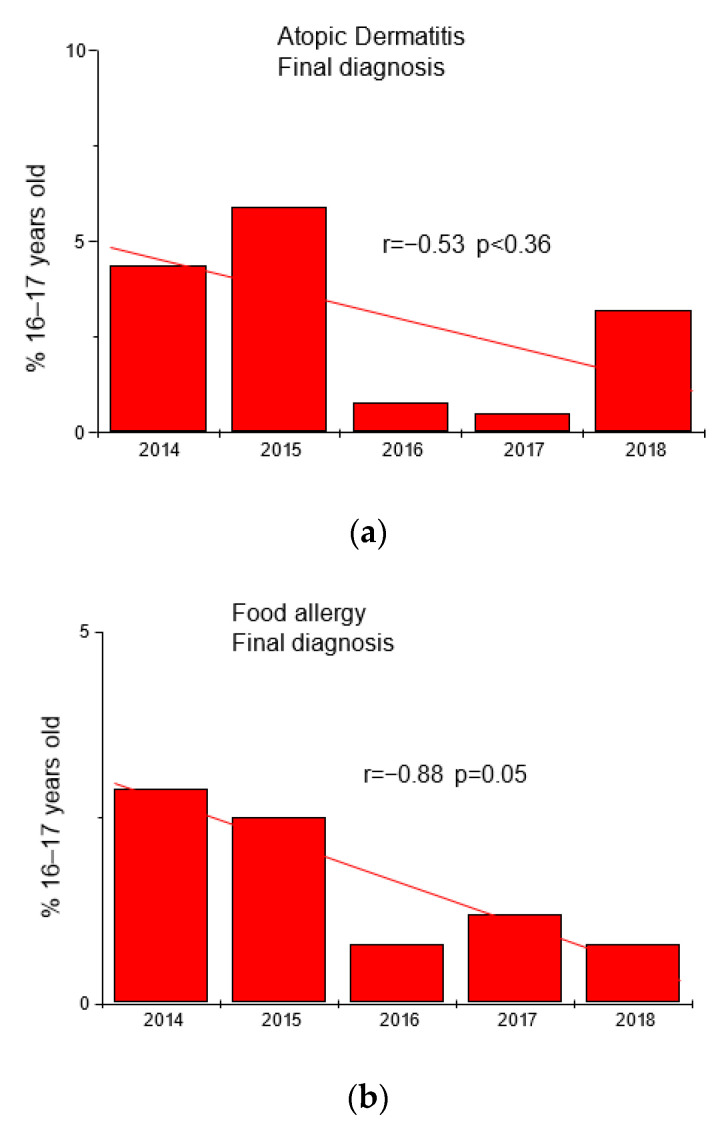
Trends in the frequency of diagnoses of atopic dermatitis (panel (**a**)) and food allergy (panel (**b**)) in the group of adolescents 16–17 years in the studied years.

Based on the results of questionnaires and interviews, additional tests were performed in 763 adolescents (fx5 panel with food allergens). The results are shown in Table 2 and Figure 2.

**Figure 3:**
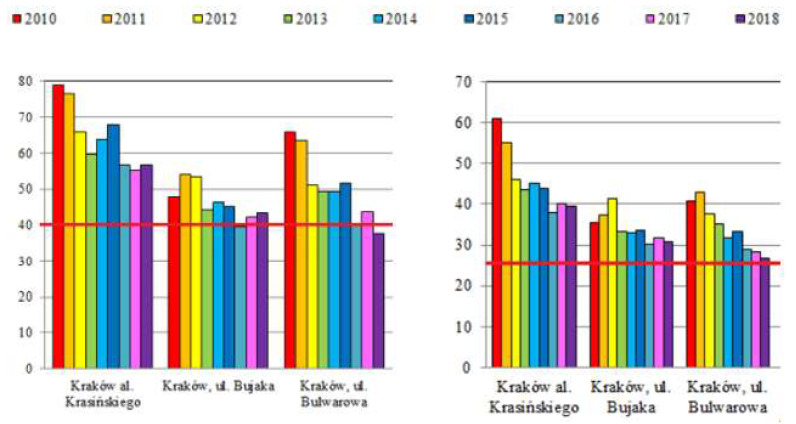
Annual mean concentrations of PM10 (**left**) and PM2.5 (**right**) in Krakow from three measurement points (µg/m^3^) in the years 2010–2018 according to Chief Inspectorate For Environmental Protection in Poland (modified); the norm value (40 µg/m^3^ for PM10 and 25 µg/m^3^ for PM2.5) is marked with a red line [11].

Data on PM concentration in the air in Krakow and their correlation with the diagnosis of AD and FA are shown in Figure 3 and Table 3.

## 4. Discussion

Atopic dermatitis is a chronic or chronically relapsing inflammatory skin disease often co-occurring with other atopic diseases (bronchial asthma and/or allergic rhinoconjunctivitis). AD is one of the most common non-transmissible skin diseases that affects up to 20% of children and 2–8% of adults in most countries around the world. AD often begins in childhood, with severe cases possibly persisting in adulthood. About one third of adult cases starts anew. AD is frequently followed by other atopic conditions, such as allergic rhinoconjunctivitis, asthma and food allergy (FA) [12].

Food allergy has been described as adverse reactions to food in which ‘immunologic mechanisms have been shown’ encompassing both immunoglobulin E (IgE)-mediated and non-IgE-mediated food allergies [13]. FA can result in considerable morbidity and in part results in life-threatening anaphylaxis.

As our research results show, the prevalence of FA in the studied time range increased in the younger age group; therefore, it cannot explain the decreasing prevalence of new cases of atopic dermatitis in this age group. The situation is different in the older age group, where the prevalence of FA decreased. We can suspect that this is the result of children growing out of an FA. It may be also influenced by more consequential eating habits in the group of adolescents and elimination of allergenic foods from the diet.

In contrast, the incidence of AD appears to be related to exposure to polluted air. In addition to the improvement of air quality in Krakow [11] (Table 3), we could observe a trend of decreasing AD frequency (Figure 3). As shown by the results of our previous studies on asthma and allergic rhinitis, the trend apparently has reversed for some diseases [14].

To understand how climate change affects children and adolescent health and well-being is an exigent matter. As demonstrated, the morbidity and mortality of many health conditions, including allergic diseases, will accelerate or worsen due to prenatal and postnatal exposure to ambient air pollutants [15,16,17,18]. Ambient air pollutants change the microbiota milieu, alter the immune response, and have a direct impact on the skin and respiratory epithelium, which facilitates the penetration of allergens [15].

Air pollutants can have perilous effects on human health. Existing in either particulate or gaseous form, they are classified as primary or secondary pollutants depending on their sources of incidence. Main primary pollutants include sulfur oxide compounds, nitrogen oxide compounds, carbon monoxide, volatile organic compounds, particulate matter (PM) and toxic metals. Secondary pollutants formed from primary pollutants in the atmosphere include ground level ozone, nitrogen dioxide and sulfuric acid [19].

PM (solid and liquid particles) can be subdivided by its aerodynamic diameter into the following categories: PM10 (<10 mm), PM2.5 (<2.5 mm), and PM0.1 (ultra-fine particulates; <0.1 mm). PM harmfully affects the airways, and has a significant influence on the respiratory system [20].

Unfortunately, 80% of individuals living in metropolitan areas are exposed to air pollution levels that exceed WHO guidelines [21].

It has been recently shown that indoor exposure to PM2.5 exacerbated AD symptoms in children, particularly children with inhalant allergies and severe symptoms. Minimizing exposure to indoor PM2.5 is essential for the proper management of AD [22].

A significant association between exposure to air pollutants (benzene, PM10, nitrogen oxide compounds, and CO exposure) and eczema symptoms was revealed in a study with 9–11-year-old children in France [23]. In Taiwan, a survey involving over 30,000 children indicated that flexural eczema incidence was associated with exposure to traffic-related air pollutants (TRAP) [24]. A birth cohort study in an urban area showed strong positive relationships between the distance to the nearest main road and eczema, while NO_2_ exposure was positively associated with eczema incidence [25]. Outdoor air pollution influences the prevalence of AD. In a study involving 4907 children, the lifetime prevalence of AD eczema was demonstrated to be significantly associated with long-term averaged concentrations of PM10, nitrogen oxide compounds, and CO [26]. The results of a study conducted in Italy provide evidence that air pollution has a significant impact on skin reactivity and symptoms in AD patients, increasing its severity [27]. These studies suggest that outdoor air pollution is one of the most potent risk factors for the development of AD and it also affects the skin of AD patients [19].

We know from a prospective study that AD patients had symptoms on days when the concentrations of outdoor PM10, PM2.5, toluene, and total volatile organic compounds were higher than on days when they reported no symptoms [28]. Diesel exhaust particulate (DEP) accounts for most of the airborne PM in the atmosphere in large cities. In one of the studies, an increased concentration of outdoor PM10 by 1 μg/m^3^ was significantly associated with a 0.44% increase in AD symptoms on the following day [28]. The effect of PM on AD was also investigated in a study in which daily symptom scores and daily PM concentrations were measured in school children aged 8 to 12 years [26]. The pruritus score was significantly associated with PM concentrations below 0.1μm, but this was not observed for larger particles [29].

Research from the Canadian Healthy Infant Longitudinal Development (CHILD) Study involving families and their infants showed that TRAP exposure during the first year of life was associated with increased risk of atopy [30].

The study showed that sensitivity to allergens was associated with exposure to TRAP during infancy, finding a link between air pollution and measured allergic sensitization during the first year of life [30].

The mechanism by which air pollution contributes to the development and exacerbation of atopic dermatitis is still uncertain. An experimental study on AD animal models documented an aggravation of the patients’ skin condition when exposed to PM10, leading to an increase in clinical severity scores, transepithelial water loss (TEWL), and epidermal thickness. PM10 induced/intensified skin inflammation due to the differential expression of genes controlling the integrity of the skin barrier and immune response [31].

## 5. Conclusions

The conclusion is that although living in a city is a risk factor for atopic dermatitis, as shown in previous research conducted in Poland and worldwide, the trend may be reversed. In our study, we were able to show a reduction in the incidence of atopic dermatitis over the 10 years analyzed, which, as we suspect in the context of the declining number of cases of food allergy, can be associated with an improvement in air quality.

Understanding that environmental exposures affect the development of allergic diseases can help us to undertake appropriate preventative measures. Reducing exposure to air pollution can be a protective factor in the development of atopic dermatitis.

## 6. Strengths and Limitations

An undoubted advantage of the presented paper is the fact that it is based on a large research group, which is the strength of the study in both the national and European context. So far, only a few studies have been conducted in Poland. We were able to analyze the occurrence of AD and FA and their variability over time in the population of children and adolescents living in one of the largest Polish cities and compare them with data on exposure to air pollution in the studied period of time. What may be considered as a limitation of the paper, and certainly requires further research, is the relationship between the incidence of atopic dermatitis and FA with exposure to polluted air, which is difficult to assess directly in humans, and is based on the correlation of compared parameters with many values that must be monitored and potential bias that must be avoided, especially in the study design.

## Figures and Tables

**Table 1:** Final diagnoses of atopic dermatitis (AD) and food allergy (FA) in the group of children 7–8 years.

Year	Final Diagnosis	Number	%	Final Diagnosis	Number	%
2014	AD	58	16.5	FA	20	5.7
2015	AD	68	18.3	FA	14	3.8
2016	AD	57	13.8	FA	21	5.1
2017	AD	44	9.2	FA	34	7.1
2018	AD	62	13.8	FA	31	6.9

**Table 2:** Final diagnoses of atopic dermatitis (AD) and food allergy (FA) in the group of adolescents 16–17 years.

Year	Final Diagnosis	Number	%	Final Diagnosis	Number	%
2014	AD	6	4.4	FA	4	2.9
2015	AD	7	5.9	FA	3	2.5
2016	AD	2	0.8	FA	2	0.8
2017	AD	8	0.5	FA	2	1.2
2018	AD	4	3.2	FA	1	0.8

**Table 3:** Analysis of the correlation between the diagnosis of atopic dermatitis (AD) and food allergy (FA) and the concentration of PM10 and PM2.5 in the measurement points in the studied years.

Correlation Coefficient	AD 7–8	AD 16–17	FA 7–8	FA 16–17
PM 10 Krasinskiego	0.905; *p* = 0.034	0.919; *p* = 0.027	−0.821; *p* = 0.088	0.838; *p* = 0.076
PM 10 Bujaka	0.903; *p* = 0.036	0.630; *p* = 0.255	−0.855; *p* = 0.065	0.366; *p* = 0.545
PM 10 Bulwarowa	0.649; *p* = 0.236	0.733; *p* = 0.159	−0.665; *p* = 0.221	0.935; *p* = 0.020
PM 2.5 Krasinskiego	0.608; *p* = 0.277	0.744; *p* = 0.150	−0.366; *p* = 0.544	0.986; *p* = 0.002
PM 2.5 Bujaka	0.630; *p* = 0.255	0.225; *p* = 0.716	−0.827; *p* = 0.084	0.144; *p* = 0.818
PM 2.5 Bulwarowa	0.725; *p* = 0.165	0.654; *p* = 0.231	−0.862; *p* = 0.060	0.832; *p* = 0.080

## Data Availability

Publicly available datasets were analyzed in this study. These data can be found here: http://powietrze.gios.gov.pl/pjp/documents/download/103138 (accessed on 18 December 2021).
